# The Week's Parliament and Publications

**Published:** 1910-07-09

**Authors:** 


### THE WEEK'S PARLIAMENT AND PUBLICATIONS.

### THE MEDICAL DEPARTMENT OF THE NAVY.

MR. MCKENNA has informed Lord Charles Beresford that the preparation of a Blue Book containing such portions of the Report as relate to the Medical Department of the Navy will be proceeded with when certain financial considerations which are involved are settled.

### HOSPITALS IN INDIA.

THE report on the moral and material progress and condition of India during the year 1908-09, which has just been issued, states that the civil hospitals and dispensaries in India, which numbered 2,593 in 1908, against 2,514 in 1907, treated 443,909 indoor and 26,631,945 outdoor patients during the year. The great bulk of their income is derived from Government and local and municipal funds, and their total expenditure in 1908 was £812,700. In addition, there is a large number of special public and other medical institutions, as well as many private institutions, which are not State-aided. These numbered 1,476, and provided treatment for 142,343 indoor and 6,507,014 outdoor patients.

### LEAD POISONING.

THE Report of the Departmental Committee appointed to consider the dangers attendant on the use of lead in the various branches of the manufacture of china and earthenware, and in the processes incidental thereto, has been issued. The regulations proposed by the Committee are stringent, and it is obvious that the much-needed reforms cannot be introduced without considerable expense, which may appreciably raise the cost of pottery in this country. It is proposed to give the certifying factory surgeon of the district power of suspension and of permission to work as regards all persons examined by him in pursuance of the regulations.—*Committee on Lead, etc., in Potteries,* Vol. i. (Cd. 5219). Price 1s. 5d.

### MEDICAL MEN AND THE PETROL TAX.

THE Chancellor of the Exchequer has been asked whether members of the medical profession who desire to claim the rebate of 1½d. per gallon upon the petrol used by them in their practice are required to attend at the office of an officer of Customs and Excise in order to sign the requisite declaration and produce the proofs necessary to satisfy the officer. Mr. Hobhouse, replying on behalf of Mr. Lloyd George, said that as a general rule it was thought desirable that applications for rebate of motor spirit should be signed in the presence of an official of the Customs and Excise Department, but officers are instructed not to insist upon this requirement in any case in which it would entail inconvenience to a claimant and where there is no doubt as to the correctness of the claim.

### MEDICAL INSPECTION OF SCHOOLS.

MR. RUNCIMAN has informed Mr. Ramsay MacDonald that the Board of Education decided to postpone the operation of the regulation which provided that children attending school should be medically examined when they reached the age of seven, in order to enable local education authorities to systematise the work they are at present doing. A number of authorities are undertaking the medical inspection of children of all ages suffering from particular ailments, and the Board are unwilling to interrupt this work. A minute of the Board of Education dated June 25, 1910, modifying the regulations for public elementary schools, has been issued. Article 58 (*b*) in the regulations, which came into operation on August 1, 1909, is to be replaced by the following: "The Board must be satisfied that provision has been made for the medical inspection of all children admitted to the school in the year, and of all children who are expected to leave school in the year—the year in each case being the twelve months ending on July 31."

### THE SPIRIT DUTY.

THE hope had been entertained that the Finance Bill would make provision for the remission of the extra duty on spirit imposed last year, but this hope has been disappointed, and the full tax is to be retained. It now remains for the Customs and Excise authorities to renew their efforts to discover some practical method of distinguishing between spirit taken out of bond for potable purposes and spirit for use in medicine. The extra duty of 3s. 9d. per proof gallon imposed last year was equivalent to a duty of 5s. 11½d. per gallon of rectified spirit, and as the previous duty on a gallon of rectified spirit was 17s. 7d. per gallon, every gallon of rectified spirit used in the preparation of medicines costs 23s. 6½d. more than it would if its use in medicine were allowed free of duty. All previous attempts to obtain a remission of any part of the tax in favour of spirituous medicinal preparations have been unsuccessful, but this is not a reason why further representations should not be made to the Chancellor of the Exchequer. And the case for hospitals would be stronger if a remission of *the whole of the tax* were requested. Hitherto the difficulty has been to devise some means of following up spirit taken out of bond for the manufacture of medicines; Mr. Goschen in 1890, Sir William Harcourt in 1894, and Sir Michael Hicks Beach in 1900 gave this as their reason for their inability to allow any rebate of the tax on medicinal spirit. Mr. Lloyd George experiences the same difficulty. The prices of between three and four hundred pharmacopœial and non-official preparations are affected by the spirit duty, and between 20 and 25 per cent. of a hospital's expenditure on drugs is in respect of this duty.

